# 1-{2-[(*E*)-2-(2-Nitro­phen­yl)ethen­yl]-1-phenyl­sulfonyl-1*H*-indol-3-yl}ethanone

**DOI:** 10.1107/S1600536813022241

**Published:** 2013-08-14

**Authors:** S. Karthikeyan, K. Sethusankar, Velu Saravanan, Arasambattu K. Mohanakrishnan

**Affiliations:** a Department of Physics, RKM Vivekananda College (Autonomous), Chennai 600 004, India; b Department of Organic Chemistry, University of Madras, Maraimalai Campus, Chennai 600 025, India

## Abstract

In the title compound, C_24_H_18_N_2_O_5_S, the S atom has a distorted tetra­hedral configuration, with bond angles varying from 105.11 (7) to 119.98 (8)°. As a result of the electron-withdrawing character of the phenyl­sulfonyl group, the N—C*sp* ^2^ bond lengths [1.414 (2) and 1.413 (2) Å] are slightly longer than the reported value of 1.355 (14) Å for N atoms with a planar configuration. The indole moiety is essentially planar, with a maximum deviation of 0.0177 (14) Å for the N atom. The phenyl ring of the sulfonyl substituent makes a dihedral angle of 85.70 (7)° with the mean plane of the indole moiety. The mol­ecular structure features intra­molecular C—H⋯O hydrogen bonds, which generate *S*(6) and *S*(12) ring motifs. In the crystal, adjacent mol­ecules are linked *via* C—H⋯O hydrogen bonds, forming infinite *C*(7) chains running along the *a*-axis direction. The crystal packing also features C—H⋯π inter­actions, which form a three-dimensional structure.

## Related literature

For the biological activity of Indole derivatives, see: Rodriguez *et al.* (1985 ▶); Chai *et al.* (2006 ▶); Olgen & Coban (2003 ▶). For related crystal structures, see: Karthikeyan *et al.* (2011 ▶, 2012 ▶). For related bond distances and bond-angle geometries and distortions, see: Allen (1981 ▶); Allen *et al.* (1987 ▶). For graph-set notation, see: Bernstein *et al.* (1995 ▶). For the Thorpe–Ingold effect, see: Bassindale (1984 ▶).
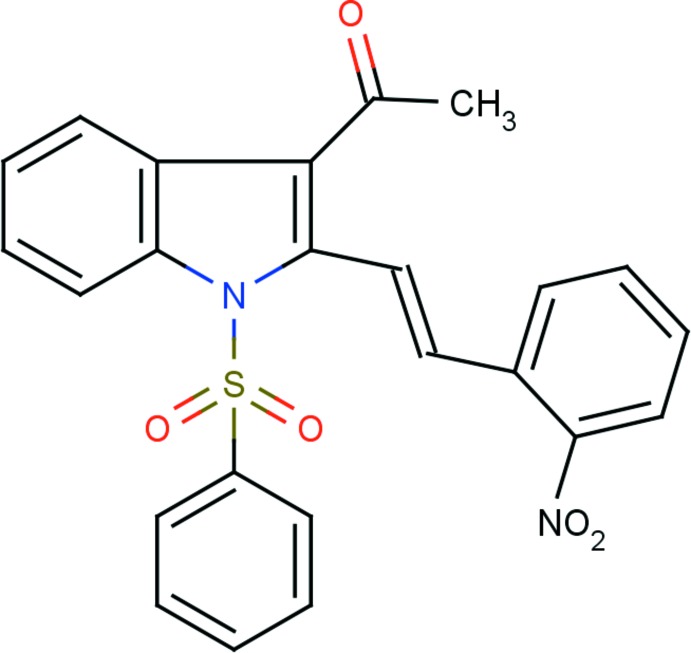


## Experimental

### 

#### Crystal data

C_24_H_18_N_2_O_5_S
*M* *_r_* = 446.46
Monoclinic, 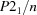

*a* = 8.2409 (8) Å
*b* = 16.1702 (15) Å
*c* = 15.3700 (15) Å
β = 95.775 (5)°
*V* = 2037.8 (3) Å^3^
*Z* = 4
Mo *K*α radiation
μ = 0.20 mm^−1^
*T* = 296 K
0.28 × 0.25 × 0.23 mm

#### Data collection

Bruker SMART APEXII CCD diffractometer
18837 measured reflections
4960 independent reflections
3893 reflections with *I* > 2σ(*I*)
*R*_int_ = 0.021

#### Refinement

*R*[*F* ^2^ > 2σ(*F* ^2^)] = 0.041
*wR*(*F* ^2^) = 0.118
*S* = 1.02
4960 reflections
290 parameters
H-atom parameters constrained
Δρ_max_ = 0.42 e Å^−3^
Δρ_min_ = −0.38 e Å^−3^

### 

Data collection: *APEX2* (Bruker, 2008 ▶); cell refinement: *SAINT* (Bruker, 2008 ▶); data reduction: *SAINT*; program(s) used to solve structure: *SHELXS97* (Sheldrick, 2008 ▶); program(s) used to refine structure: *SHELXL97* (Sheldrick, 2008 ▶); molecular graphics: *ORTEP-3 for Windows* (Farrugia, 2012 ▶) and *Mercury* (Macrae *et al.*, 2008 ▶); software used to prepare material for publication: *SHELXL97* and *PLATON* (Spek, 2009 ▶).

## Supplementary Material

Crystal structure: contains datablock(s) global, I. DOI: 10.1107/S1600536813022241/su2631sup1.cif

Structure factors: contains datablock(s) I. DOI: 10.1107/S1600536813022241/su2631Isup2.hkl

Supplementary material file. DOI: 10.1107/S1600536813022241/su2631Isup3.cml

Additional supplementary materials:  crystallographic information; 3D view; checkCIF report

## Figures and Tables

**Table 1:** Hydrogen-bond geometry (Å, °) *Cg*1, *Cg*2 and *Cg*3 are the centroids of the C17–C22, C11–C16 and C1–C6 rings, respectively.

*D*—H⋯*A*	*D*—H	H⋯*A*	*D*⋯*A*	*D*—H⋯*A*
C2—H2⋯O1	0.93	2.35	2.935 (2)	121
C18—H18⋯O3	0.93	2.46	3.108 (2)	127
C20—H20⋯O2^i^	0.93	2.68	3.319 (2)	126
C5—H5⋯*Cg*1^ii^	0.93	2.89	3.720 (2)	149
C22—H22⋯*Cg*2^iii^	0.93	2.73	3.4618 (18)	137
C24—H24*B*⋯*Cg*3^iv^	0.96	2.90	3.601 (3)	131

Symmetry codes: (i) 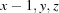; (ii) 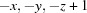; (iii) 
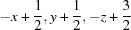
; (iv) 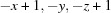.

**Fig. 1.**
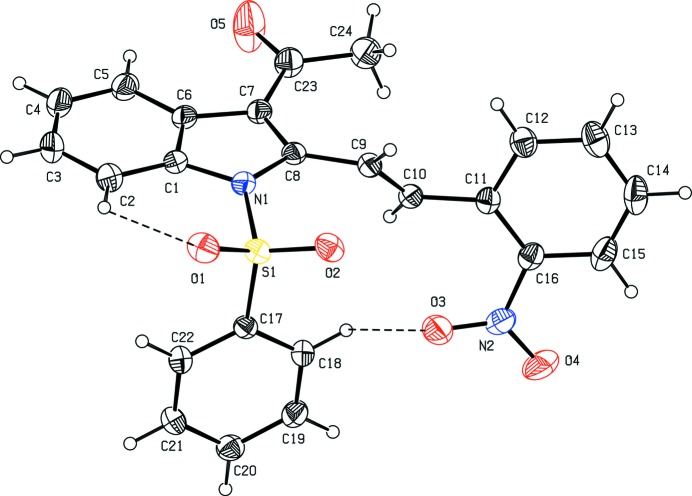
The molecular structure of the title molecule, with the atom labelling. Displacement ellipsoids are drawn at 30% probability level. The intramolecular C-H···O hydrogen bonds are shown as dashed lines (see Table 1 for details).

**Fig. 2.**
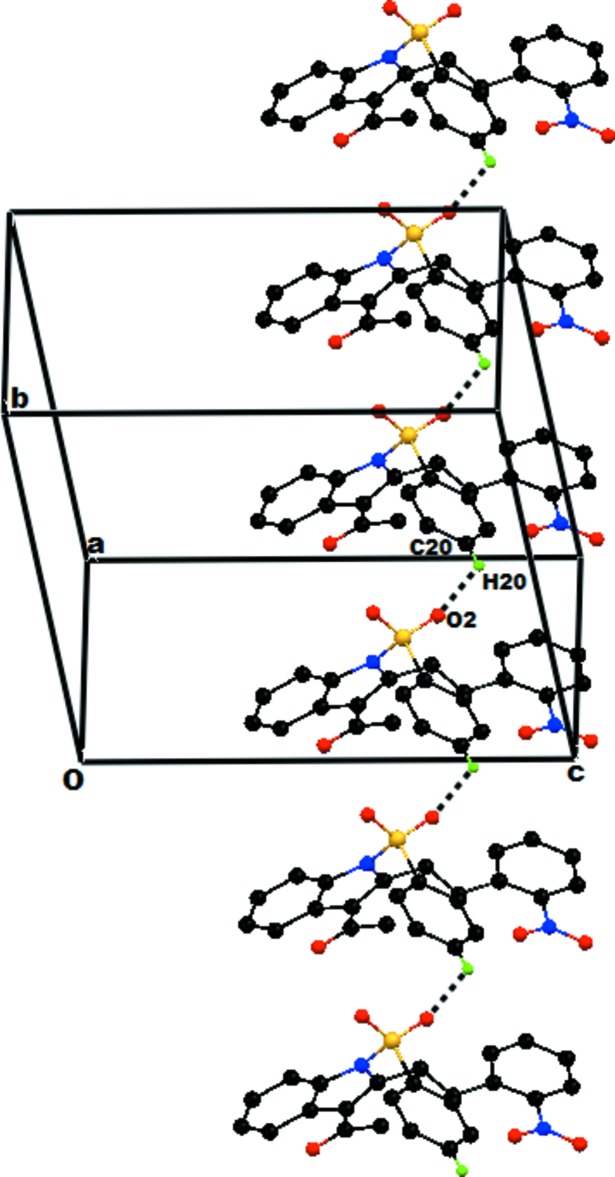
The crystal packing of the title compound viewed along the a axis, showing the formation of infinite C(7) chains. The dashed lines indicate C—H···O hydrogen bonds (see Table 1 for details).

**Fig. 3.**
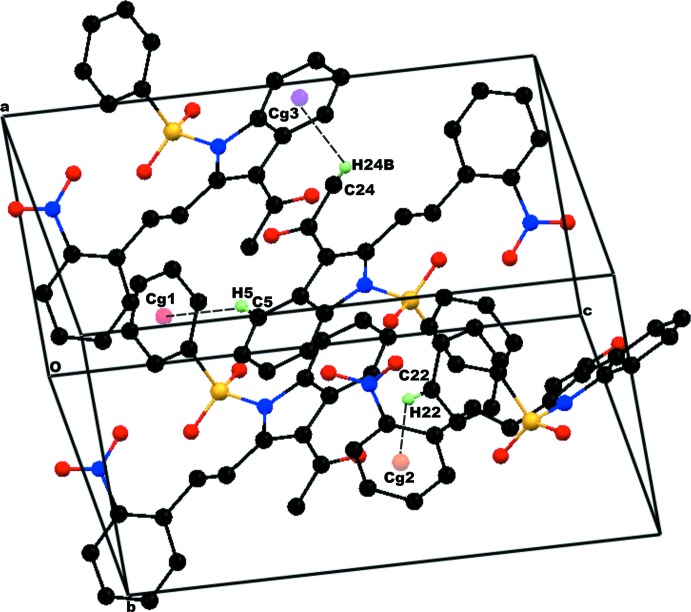
The crystal packing of the title compound, showing the intermolecular C—H···π interactions as dashed lines (centroid = red sphere; see Table 1 for details).

## supplementary crystallographic information

### 1. Comment

The indole ring system is present in a number of natural products, many of which are found to possess pharmacological properties like anti-microbial, anti-inflammatory and anti-implantation activities (Rodriguez *et al.*, 1985). Indole derivatives are also known to exhibit anti-oxidant activities (Olgen & Coban, 2003) and antihepatitis B virus activities (Chai *et al.*, 2006).

In the title compound, Fig. 1, the indole ring is essentially planar with a maximum deviation of 0.0177 (14) Å for atom N1. The indole moiety is perpendicular to the phenylsulfonyl ring with a dihedral angle 85.70 (7)°. The nitro-group is inclined at an angle of 7.59 (9)° to the benzene ring to which it is attached. The nitro-phenyl ring makes a dihedral angle of 76.41 (7)° with the indole moiety mean plane. Short intramolecular C2—H2···O1 and C18—H18···O3 hyrogen bonds result in *S*(6) and *S*(12) ring motifs (Table 1 and Fig. 1). The molecular dimensions in the title compound are in excellent agreement with those reported for a closely related compound (Karthikeyan *et al.*, 2012).

The expansion of the ispo angles at C1, C3 and C4 [122.34 (16), 121.59 (18) and 121.13 (18)°, respectively] and contraction of the apical angles at C2, C5 and C6 [117.12 (19), 118.54 (18) and 119.28 (16)°, respectively] are caused by the fusion of the smaller pyrrole ring to the six-membered benzene ring and the strain is taken up by the angular distortion rather than by bond-length distortions (Allen, 1981). As a result of the electron withdrawing character of the phenylsulfonyl group, the N—C*sp*^2^ bond lengths, *viz*, N1—C1 [1.414 (2) Å] and N1—C8 [1.413 (2) Å], are longer than the mean value of 1.355 (14) Å reported for N atoms with planar configurations (Allen *et al.*, 1987).

Atom S1 has a distorted tetrahedral configuration. The widening of the angle O2═S1═O1 [119.98 (8)°] and the narrowing of the angle N1—S1—C17 [105.11 (7)°] from the ideal tetrahedral value are attributed to the Thorpe-Ingold effect (Bassindale, 1984). The widening of the angles may be due to the repulsive interactions between the two short S═O bonds, similar to what is observed in a related structure (Karthikeyan *et al.*, 2011).

The crystal packing is stabilized by C—H···O hydrogen bonds (Fig. 2 and Table 1) resulting in *C*(7) infinite chains of molecules running along the *a* axis direction (Bernstein *et al.*, 1995). The crystal packing is further stabilized by C—H···π interactions forming a three-dimensional structure (Table 1 and Fig. 3).

### 2. Experimental

A solution of the ylide, 1-[1-(phenylsulfonyl)-2-[(triphenyl-$1^5^-phosphanylidene)methyl]-1H-indol-3-yl]ethan-1-one (1 g, 1.745 mmol), and 2-nitrobenzaldehyde (0.29 g, 1.919 mmol) in dry DCM (20 ml) was refluxed for 8 h under N_2_. Removal of solvent *in vacuo* followed by trituration of the crude product with cold methanol (5 ml) afforded the title compound as a yellow solid [Yield: 0.71 g (92%); M.p. 427-429 K]. Block-like yellow crystals were obtained by slow evaporation of a solution in CHCl_3_.

### 3. Refinement

The hydrogen atoms were localized from the difference electron density maps and were refined as riding atoms: C—H = 0.93 and 0.96 Å for CH(aromatic) and methyl H atoms, respectively, with *U*_iso_(H) = 1.5 *U*_eq_(C-methyl) and = 1.2U_eq_(C) for other H atoms. The rotation angles for the methyl groups were optimized by least squares.

### Crystal data

**Table:**

C_24_H_18_N_2_O_5_S	*F*(000) = 928
*M**_r_* = 446.46	*D*_x_ = 1.455 Mg m^−^^3^
Monoclinic, *P*2_1_/*n*	Mo *K*α radiation, λ = 0.71073 Å
Hall symbol: -p 2yn	Cell parameters from 4960 reflections
*a* = 8.2409 (8) Å	θ = 1.8–28.4°
*b* = 16.1702 (15) Å	µ = 0.20 mm^−^^1^
*c* = 15.3700 (15) Å	*T* = 296 K
β = 95.775 (5)°	Block, yellow
*V* = 2037.8 (3) Å^3^	0.28 × 0.25 × 0.23 mm
*Z* = 4	

### Data collection

**Table:**

Bruker SMART APEXII CCD diffractometer	3893 reflections with *I* > 2σ(*I*)
Radiation source: fine-focus sealed tube	*R*_int_ = 0.021
Graphite monochromator	θ_max_ = 28.4°, θ_min_ = 1.8°
ω and φ scans	*h* = −10→7
18837 measured reflections	*k* = −17→21
4960 independent reflections	*l* = −19→20

### Refinement

**Table:**

Refinement on *F*^2^	Primary atom site location: structure-invariant direct methods
Least-squares matrix: full	Secondary atom site location: difference Fourier map
*R*[*F*^2^ > 2σ(*F*^2^)] = 0.041	Hydrogen site location: inferred from neighbouring sites
*wR*(*F*^2^) = 0.118	H-atom parameters constrained
*S* = 1.02	*w* = 1/[σ^2^(*F*_o_^2^) + (0.059*P*)^2^ + 0.5665*P*] where *P* = (*F*_o_^2^ + 2*F*_c_^2^)/3
4960 reflections	(Δ/σ)_max_ = 0.001
290 parameters	Δρ_max_ = 0.42 e Å^−^^3^
0 restraints	Δρ_min_ = −0.38 e Å^−^^3^

### Special details

**Table:**

Geometry. All e.s.d.'s (except the e.s.d. in the dihedral angle between two l.s. planes) are estimated using the full covariance matrix. The cell e.s.d.'s are taken into account individually in the estimation of e.s.d.'s in distances, angles and torsion angles; correlations between e.s.d.'s in cell parameters are only used when they are defined by crystal symmetry. An approximate (isotropic) treatment of cell e.s.d.'s is used for estimating e.s.d.'s involving l.s. planes.
Refinement. Refinement of *F*^2^ against ALL reflections. The weighted *R*-factor *wR* and goodness of fit *S* are based on *F*^2^, conventional *R*-factors *R* are based on *F*, with *F* set to zero for negative *F*^2^. The threshold expression of *F*^2^ > σ(*F*^2^) is used only for calculating *R*-factors(gt) *etc*. and is not relevant to the choice of reflections for refinement. *R*-factors based on *F*^2^ are statistically about twice as large as those based on *F*, and *R*- factors based on ALL data will be even larger.

### Fractional atomic coordinates and isotropic or equivalent isotropic displacement parameters (Å^2^)

**Table:**

	*x*	*y*	*z*	*U*_iso_*/*U*_eq_	
C1	0.21108 (19)	0.10784 (11)	0.52063 (10)	0.0405 (4)	
C2	0.1313 (3)	0.17176 (13)	0.47358 (13)	0.0570 (5)	
H2	0.1284	0.2250	0.4963	0.068*	
C3	0.0567 (2)	0.15297 (15)	0.39184 (13)	0.0627 (5)	
H3	0.0019	0.1944	0.3587	0.075*	
C4	0.0611 (2)	0.07382 (14)	0.35767 (12)	0.0579 (5)	
H4	0.0089	0.0631	0.3023	0.070*	
C5	0.1414 (2)	0.01077 (13)	0.40421 (11)	0.0499 (4)	
H5	0.1450	−0.0421	0.3806	0.060*	
C6	0.21746 (19)	0.02793 (11)	0.48764 (10)	0.0395 (4)	
C7	0.31131 (18)	−0.02214 (10)	0.55255 (10)	0.0379 (3)	
C8	0.35638 (18)	0.02700 (9)	0.62314 (10)	0.0356 (3)	
C9	0.45622 (19)	0.00804 (10)	0.70516 (10)	0.0375 (3)	
H9	0.5562	0.0346	0.7165	0.045*	
C10	0.41139 (19)	−0.04492 (10)	0.76375 (10)	0.0381 (3)	
H10	0.3066	−0.0668	0.7559	0.046*	
C11	0.5202 (2)	−0.07076 (9)	0.84085 (10)	0.0386 (3)	
C12	0.6855 (2)	−0.08209 (11)	0.83104 (13)	0.0500 (4)	
H12	0.7229	−0.0696	0.7775	0.060*	
C13	0.7946 (2)	−0.11089 (13)	0.89725 (15)	0.0618 (5)	
H13	0.9040	−0.1172	0.8885	0.074*	
C14	0.7415 (3)	−0.13056 (13)	0.97719 (14)	0.0652 (6)	
H14	0.8147	−0.1514	1.0218	0.078*	
C15	0.5818 (3)	−0.11937 (12)	0.99067 (12)	0.0566 (5)	
H15	0.5464	−0.1316	1.0448	0.068*	
C16	0.4726 (2)	−0.08979 (10)	0.92347 (10)	0.0422 (4)	
C17	0.10318 (18)	0.16933 (9)	0.72059 (10)	0.0339 (3)	
C18	0.0997 (2)	0.11710 (11)	0.79151 (10)	0.0426 (4)	
H18	0.1937	0.0897	0.8143	0.051*	
C19	−0.0445 (2)	0.10613 (12)	0.82802 (11)	0.0478 (4)	
H19	−0.0483	0.0711	0.8757	0.057*	
C20	−0.1833 (2)	0.14712 (12)	0.79391 (11)	0.0460 (4)	
H20	−0.2800	0.1404	0.8195	0.055*	
C21	−0.1796 (2)	0.19756 (12)	0.72260 (12)	0.0466 (4)	
H21	−0.2743	0.2241	0.6994	0.056*	
C22	−0.03571 (19)	0.20929 (10)	0.68482 (11)	0.0415 (4)	
H22	−0.0328	0.2434	0.6363	0.050*	
C23	0.3572 (2)	−0.10893 (11)	0.53594 (12)	0.0513 (4)	
C24	0.4614 (3)	−0.15953 (12)	0.59949 (13)	0.0617 (5)	
H24A	0.3984	−0.1781	0.6450	0.093*	
H24B	0.5515	−0.1268	0.6245	0.093*	
H24C	0.5018	−0.2065	0.5702	0.093*	
N1	0.30044 (16)	0.10825 (8)	0.60412 (8)	0.0394 (3)	
N2	0.3042 (2)	−0.07812 (9)	0.94350 (10)	0.0501 (4)	
O1	0.28218 (16)	0.25924 (7)	0.62838 (9)	0.0549 (3)	
O2	0.41828 (14)	0.17130 (8)	0.74404 (8)	0.0498 (3)	
O3	0.20813 (17)	−0.04423 (10)	0.89080 (9)	0.0653 (4)	
O4	0.2671 (2)	−0.10333 (12)	1.01319 (10)	0.0862 (5)	
O5	0.3144 (3)	−0.13858 (12)	0.46632 (13)	0.1255 (10)	
S1	0.28946 (5)	0.18452 (2)	0.67705 (3)	0.03893 (12)	

### Atomic displacement parameters (Å^2^)

**Table:**

	*U*^11^	*U*^22^	*U*^33^	*U*^12^	*U*^13^	*U*^23^
C1	0.0387 (8)	0.0493 (9)	0.0345 (8)	0.0064 (7)	0.0078 (6)	0.0042 (7)
C2	0.0639 (12)	0.0574 (11)	0.0499 (10)	0.0185 (9)	0.0072 (9)	0.0088 (9)
C3	0.0602 (12)	0.0790 (14)	0.0479 (11)	0.0200 (11)	0.0012 (9)	0.0180 (10)
C4	0.0465 (10)	0.0853 (15)	0.0409 (10)	0.0039 (10)	−0.0008 (8)	0.0064 (9)
C5	0.0443 (9)	0.0639 (11)	0.0414 (9)	−0.0026 (8)	0.0030 (7)	−0.0025 (8)
C6	0.0354 (8)	0.0484 (9)	0.0356 (8)	−0.0002 (7)	0.0080 (6)	0.0027 (7)
C7	0.0364 (8)	0.0411 (8)	0.0367 (8)	−0.0010 (6)	0.0060 (6)	0.0015 (6)
C8	0.0336 (7)	0.0380 (8)	0.0363 (8)	0.0016 (6)	0.0089 (6)	0.0037 (6)
C9	0.0354 (8)	0.0392 (8)	0.0378 (8)	0.0019 (6)	0.0028 (6)	−0.0007 (6)
C10	0.0350 (8)	0.0430 (9)	0.0363 (8)	0.0012 (6)	0.0033 (6)	−0.0008 (7)
C11	0.0429 (9)	0.0342 (8)	0.0376 (8)	−0.0022 (6)	−0.0006 (6)	−0.0016 (6)
C12	0.0441 (9)	0.0502 (10)	0.0547 (10)	−0.0022 (8)	0.0004 (8)	0.0038 (8)
C13	0.0457 (10)	0.0613 (12)	0.0743 (14)	−0.0011 (9)	−0.0142 (9)	−0.0001 (10)
C14	0.0725 (14)	0.0595 (12)	0.0569 (12)	0.0018 (10)	−0.0271 (10)	0.0017 (10)
C15	0.0834 (15)	0.0468 (10)	0.0369 (9)	−0.0011 (9)	−0.0081 (9)	−0.0002 (8)
C16	0.0551 (10)	0.0337 (8)	0.0370 (8)	−0.0014 (7)	0.0014 (7)	−0.0035 (6)
C17	0.0335 (7)	0.0331 (7)	0.0347 (7)	−0.0002 (6)	0.0020 (6)	−0.0054 (6)
C18	0.0422 (9)	0.0481 (9)	0.0366 (8)	0.0067 (7)	−0.0005 (7)	0.0026 (7)
C19	0.0499 (10)	0.0556 (10)	0.0382 (9)	0.0001 (8)	0.0066 (7)	0.0065 (8)
C20	0.0377 (9)	0.0561 (10)	0.0446 (9)	−0.0045 (7)	0.0062 (7)	−0.0037 (8)
C21	0.0349 (8)	0.0533 (10)	0.0506 (10)	0.0052 (7)	−0.0008 (7)	0.0015 (8)
C22	0.0417 (9)	0.0401 (8)	0.0419 (9)	0.0022 (7)	−0.0007 (7)	0.0050 (7)
C23	0.0586 (11)	0.0445 (10)	0.0495 (10)	−0.0001 (8)	−0.0006 (8)	−0.0066 (8)
C24	0.0823 (15)	0.0428 (10)	0.0596 (12)	0.0124 (10)	0.0054 (10)	−0.0016 (9)
N1	0.0437 (7)	0.0401 (7)	0.0349 (7)	0.0076 (6)	0.0060 (5)	0.0013 (5)
N2	0.0696 (10)	0.0408 (8)	0.0419 (8)	0.0020 (7)	0.0159 (7)	0.0004 (6)
O1	0.0575 (8)	0.0386 (6)	0.0708 (8)	−0.0015 (5)	0.0165 (6)	0.0090 (6)
O2	0.0369 (6)	0.0521 (7)	0.0589 (8)	−0.0016 (5)	−0.0030 (5)	−0.0118 (6)
O3	0.0606 (9)	0.0722 (10)	0.0661 (9)	0.0154 (7)	0.0206 (7)	0.0209 (7)
O4	0.1070 (13)	0.1054 (13)	0.0524 (9)	0.0156 (11)	0.0377 (9)	0.0191 (9)
O5	0.191 (2)	0.0768 (12)	0.0917 (13)	0.0510 (14)	−0.0689 (14)	−0.0432 (11)
S1	0.0365 (2)	0.0349 (2)	0.0456 (2)	−0.00095 (15)	0.00548 (16)	−0.00227 (16)

### Geometric parameters (Å, º)

**Table:**

C1—C2	1.389 (2)	C14—H14	0.9300
C1—C6	1.391 (2)	C15—C16	1.385 (2)
C1—N1	1.414 (2)	C15—H15	0.9300
C2—C3	1.376 (3)	C16—N2	1.464 (2)
C2—H2	0.9300	C17—C22	1.380 (2)
C3—C4	1.385 (3)	C17—C18	1.382 (2)
C3—H3	0.9300	C17—S1	1.7526 (15)
C4—C5	1.376 (3)	C18—C19	1.375 (2)
C4—H4	0.9300	C18—H18	0.9300
C5—C6	1.397 (2)	C19—C20	1.379 (2)
C5—H5	0.9300	C19—H19	0.9300
C6—C7	1.447 (2)	C20—C21	1.369 (3)
C7—C8	1.366 (2)	C20—H20	0.9300
C7—C23	1.482 (2)	C21—C22	1.385 (2)
C8—N1	1.413 (2)	C21—H21	0.9300
C8—C9	1.467 (2)	C22—H22	0.9300
C9—C10	1.322 (2)	C23—O5	1.193 (2)
C9—H9	0.9300	C23—C24	1.480 (3)
C10—C11	1.473 (2)	C24—H24A	0.9600
C10—H10	0.9300	C24—H24B	0.9600
C11—C12	1.397 (2)	C24—H24C	0.9600
C11—C16	1.401 (2)	N1—S1	1.6752 (14)
C12—C13	1.370 (3)	N2—O3	1.2058 (19)
C12—H12	0.9300	N2—O4	1.2135 (19)
C13—C14	1.382 (3)	O1—S1	1.4192 (13)
C13—H13	0.9300	O2—S1	1.4187 (12)
C14—C15	1.365 (3)		
			
C2—C1—C6	122.34 (16)	C16—C15—H15	120.1
C2—C1—N1	130.19 (17)	C15—C16—C11	122.11 (17)
C6—C1—N1	107.45 (13)	C15—C16—N2	116.51 (16)
C3—C2—C1	117.12 (19)	C11—C16—N2	121.38 (15)
C3—C2—H2	121.4	C22—C17—C18	121.22 (15)
C1—C2—H2	121.4	C22—C17—S1	120.38 (12)
C2—C3—C4	121.59 (18)	C18—C17—S1	118.40 (12)
C2—C3—H3	119.2	C19—C18—C17	119.23 (15)
C4—C3—H3	119.2	C19—C18—H18	120.4
C5—C4—C3	121.13 (18)	C17—C18—H18	120.4
C5—C4—H4	119.4	C18—C19—C20	120.04 (16)
C3—C4—H4	119.4	C18—C19—H19	120.0
C4—C5—C6	118.54 (18)	C20—C19—H19	120.0
C4—C5—H5	120.7	C21—C20—C19	120.40 (16)
C6—C5—H5	120.7	C21—C20—H20	119.8
C1—C6—C5	119.28 (16)	C19—C20—H20	119.8
C1—C6—C7	107.80 (14)	C20—C21—C22	120.41 (16)
C5—C6—C7	132.91 (16)	C20—C21—H21	119.8
C8—C7—C6	107.81 (14)	C22—C21—H21	119.8
C8—C7—C23	129.34 (15)	C17—C22—C21	118.68 (15)
C6—C7—C23	122.62 (15)	C17—C22—H22	120.7
C7—C8—N1	108.64 (13)	C21—C22—H22	120.7
C7—C8—C9	130.18 (14)	O5—C23—C24	117.99 (18)
N1—C8—C9	121.02 (14)	O5—C23—C7	118.50 (18)
C10—C9—C8	123.36 (15)	C24—C23—C7	123.42 (16)
C10—C9—H9	118.3	C23—C24—H24A	109.5
C8—C9—H9	118.3	C23—C24—H24B	109.5
C9—C10—C11	122.86 (15)	H24A—C24—H24B	109.5
C9—C10—H10	118.6	C23—C24—H24C	109.5
C11—C10—H10	118.6	H24A—C24—H24C	109.5
C12—C11—C16	115.74 (15)	H24B—C24—H24C	109.5
C12—C11—C10	118.13 (15)	C8—N1—C1	108.22 (13)
C16—C11—C10	126.03 (15)	C8—N1—S1	125.77 (11)
C13—C12—C11	122.58 (19)	C1—N1—S1	123.54 (11)
C13—C12—H12	118.7	O3—N2—O4	122.63 (17)
C11—C12—H12	118.7	O3—N2—C16	119.31 (14)
C12—C13—C14	119.7 (2)	O4—N2—C16	118.06 (17)
C12—C13—H13	120.1	O2—S1—O1	119.98 (8)
C14—C13—H13	120.1	O2—S1—N1	106.73 (7)
C15—C14—C13	120.05 (18)	O1—S1—N1	106.04 (8)
C15—C14—H14	120.0	O2—S1—C17	108.79 (7)
C13—C14—H14	120.0	O1—S1—C17	109.17 (8)
C14—C15—C16	119.79 (19)	N1—S1—C17	105.11 (7)
C14—C15—H15	120.1		
			
C6—C1—C2—C3	0.3 (3)	S1—C17—C18—C19	178.28 (13)
N1—C1—C2—C3	178.49 (17)	C17—C18—C19—C20	−0.1 (3)
C1—C2—C3—C4	−0.2 (3)	C18—C19—C20—C21	1.3 (3)
C2—C3—C4—C5	−0.3 (3)	C19—C20—C21—C22	−1.2 (3)
C3—C4—C5—C6	0.7 (3)	C18—C17—C22—C21	1.4 (2)
C2—C1—C6—C5	0.1 (3)	S1—C17—C22—C21	−178.17 (13)
N1—C1—C6—C5	−178.46 (14)	C20—C21—C22—C17	−0.1 (3)
C2—C1—C6—C7	179.14 (16)	C8—C7—C23—O5	−174.0 (2)
N1—C1—C6—C7	0.59 (17)	C6—C7—C23—O5	−0.3 (3)
C4—C5—C6—C1	−0.6 (2)	C8—C7—C23—C24	2.5 (3)
C4—C5—C6—C7	−179.37 (18)	C6—C7—C23—C24	176.22 (17)
C1—C6—C7—C8	1.22 (17)	C7—C8—N1—C1	2.95 (17)
C5—C6—C7—C8	−179.90 (17)	C9—C8—N1—C1	178.81 (13)
C1—C6—C7—C23	−173.71 (15)	C7—C8—N1—S1	165.52 (11)
C5—C6—C7—C23	5.2 (3)	C9—C8—N1—S1	−18.6 (2)
C6—C7—C8—N1	−2.55 (17)	C2—C1—N1—C8	179.46 (18)
C23—C7—C8—N1	171.93 (16)	C6—C1—N1—C8	−2.14 (17)
C6—C7—C8—C9	−177.91 (15)	C2—C1—N1—S1	16.4 (3)
C23—C7—C8—C9	−3.4 (3)	C6—C1—N1—S1	−165.19 (11)
C7—C8—C9—C10	−65.7 (2)	C15—C16—N2—O3	172.02 (17)
N1—C8—C9—C10	119.47 (18)	C11—C16—N2—O3	−7.1 (2)
C8—C9—C10—C11	172.71 (14)	C15—C16—N2—O4	−8.0 (2)
C9—C10—C11—C12	−37.6 (2)	C11—C16—N2—O4	172.85 (17)
C9—C10—C11—C16	146.09 (17)	C8—N1—S1—O2	31.41 (15)
C16—C11—C12—C13	0.8 (3)	C1—N1—S1—O2	−168.55 (12)
C10—C11—C12—C13	−175.90 (17)	C8—N1—S1—O1	160.41 (13)
C11—C12—C13—C14	0.6 (3)	C1—N1—S1—O1	−39.55 (15)
C12—C13—C14—C15	−1.6 (3)	C8—N1—S1—C17	−84.02 (14)
C13—C14—C15—C16	1.3 (3)	C1—N1—S1—C17	76.02 (14)
C14—C15—C16—C11	0.1 (3)	C22—C17—S1—O2	152.91 (13)
C14—C15—C16—N2	−178.97 (17)	C18—C17—S1—O2	−26.65 (14)
C12—C11—C16—C15	−1.1 (2)	C22—C17—S1—O1	20.30 (15)
C10—C11—C16—C15	175.24 (16)	C18—C17—S1—O1	−159.27 (12)
C12—C11—C16—N2	177.94 (15)	C22—C17—S1—N1	−93.09 (13)
C10—C11—C16—N2	−5.7 (2)	C18—C17—S1—N1	87.35 (13)
C22—C17—C18—C19	−1.3 (2)		

### Hydrogen-bond geometry (Å, º)

Cg1, Cg2 and Cg3 are the centroids of the C17–C22, C11–C16 and C1–C6 rings, respectively.

**Table:**

*D*—H···*A*	*D*—H	H···*A*	*D*···*A*	*D*—H···*A*
C2—H2···O1	0.93	2.35	2.935 (2)	121
C18—H18···O3	0.93	2.46	3.108 (2)	127
C20—H20···O2^i^	0.93	2.68	3.319 (2)	126
C5—H5···*Cg*1^ii^	0.93	2.89	3.720 (2)	149
C22—H22···*Cg*2^iii^	0.93	2.73	3.4618 (18)	137
C24—H24*B*···*Cg*3^iv^	0.96	2.90	3.601 (3)	131

Symmetry codes: (i) *x*−1, *y*, *z*; (ii) −*x*, −*y*, −*z*+1; (iii) −*x*+1/2, *y*+1/2, −*z*+3/2; (iv) −*x*+1, −*y*, −*z*+1.
